# Encéphalopathie de Wernicke compliquant l'hyperémèse gravidique et associée à une myélinolyse centropontique

**DOI:** 10.11604/pamj.2014.19.340.5692

**Published:** 2014-12-01

**Authors:** Hanane Baouahi, Mouhssine Doumiri

**Affiliations:** 1 Département d'Anesthésie Réanimation Obstétricale, Hôpital Maternité Souissi, Université Mohamed V, Rabat, Maroc

**Keywords:** Encéphalopathie de Wernicke, myélinolyse centropontique, vomissements gravidiques, Wernicke encephalopathy, Central pontine myelinolysis, vomiting in pregnancy

## Image en medicine

Patiente âgée de 18 ans, primipare, enceinte de 12 semaines d'aménorrhées, sans antécédents, a été admise aux urgences pour prise en charge de vomissements gravidiques incoercibles depuis trois semaines. L'examen clinique a montré un subictère, des signes de déshydrations globale, une ophtalmologie, une ataxie et une confusion. Une tension artérielle systolique à 90 mmHg, une tachycardie à 126 battements par minute. la biologie a objectivé une alcalose métabolique (ph à 7.58, HCO3- à 51.7mmol/L, Bases excès à 26.7 mmol/L, PCO2 à 56mmHg, PaO2 à 83mmHg), hyponatrémie à 130mmol /L, hypokaliémie à 1.6 mmol/L, hypochlorémie à 50mmol /L, Bilirubine directe à 40mg/L, ALAT à 1977 UI UI/L, ASAT à 543 UI UI/L, Urée à 1.6g/L, Créatinine à 14 mg/L, la sérologie des hépatites virales B et C était négative, l’échographie abdominale était normale. Une Encéphalopathie de Wernicke compliquant un hyperémèse gravidique a été évoquée et confirmée par IRM cérébrale. Le traitement était à base d'une réhydration par sérum salé et glucosé, apport journalier des électrolytes, antiémétiques et vitaminothérapie à base de B1 à la dose 200mg/jour pendant un mois. L’évolution a été marquée après une semaine par l'arrêt des vomissements, retour de l’état de conscience à la normale et correction des troubles métaboliques. La patiente a quitté L'hôpital au dixième jour. Un Contrôle réalisé après 10 jours a montré des troubles de comportement. Une IRM de control a été réalisée montrant une image de myélinolyse centropentique.

**Figure 1:**
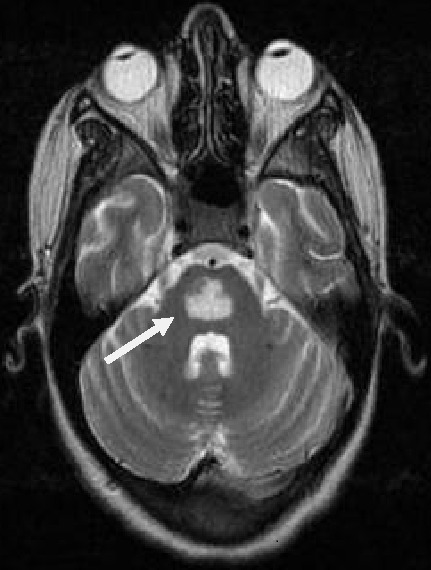
Coupe axiale en T2 la Flèche: myélinolyse centro-pentique

